# A Completely Non-occlusive Segmental Cryoballoon Ablation for Atrial Fibrillation Guided by High-density Mapping: A Novel Approach and Proof of Concept

**DOI:** 10.19102/icrm.2022.13124

**Published:** 2022-12-15

**Authors:** Kamal Kotak

**Affiliations:** ^1^Cardiac Arrhythmia Service, Department of Cardiology, Loma Linda University, Loma Linda, CA, USA

**Keywords:** Atrial fibrillation ablation, cryoballoon ablation, HD Grid, high-density mapping, segmental cryoballoon ablation

## Abstract

Cryoballoon (CB) ablation traditionally involves obtaining pulmonary vein (PV) occlusion followed by pulmonary vein isolation (PVI). The therapy is guided by time to effect and proximity to the esophagus or phrenic nerve. This, however, requires segmental non-occlusive cryoablation (NOCA) to achieve PVI. While segmental ablation has gained popularity lately with its use during left atrial posterior wall ablation, occlusive PVI remains the mainstay of CB ablation. Many times, this leads to distal lesions and not necessarily wide-area circumferential ablation (WACA) as done with radiofrequency (RF) ablation. In addition, NOCA is guided by estimated balloon positioning as there is no way to see the balloon on the mapping system or to show the exact area of balloon contact as is achievable with contact force catheters. In this case report, we show how a high-density mapping catheter can be used (1) to select the site of ablation on the WACA line; (2) to determine the anticipated location of the CB ablation lesion; (3) to ensure contact; (4) for high-density mapping to ensure PVI; (5) to avoid any PV occlusion and use of related modalities (contrast, left atrial pressure waveform, intracardiac echo, and color Doppler); (6) for short lesions to avoid any change in esophageal temperature of effect on phrenic nerve; and (7) to achieve true WACA similar to as seen with RF ablation in a very predictable manner. We believe that this is the first case report of its kind using a high-density mapping catheter without an attempt made for any PV occlusion.

## Introduction

Cryoballoon (CB) catheter ablation is a commonly used modality for the treatment of atrial fibrillation (AF). This single-shot pulmonary vein (PV) isolation (PVI) tool has several advantages, which has made it popular. However, it has become evident that lesions may not be proximal in relation to the PV ostium to achieve wide-area circumferential ablation (WACA). In addition, CB ablation requires that the operator ensures the PV seal in order to achieve PVI. At times, this can be challenging, and PVI may require additional applications—what we now refer to as non-occlusive cryoablation (NOCA).^1^ As the CB is not visible on the 3-dimensional (3D) electroanatomic mapping system, operators have to estimate the intended location for NOCA. Fluoroscopic and intracardiac echo (ICE) guidance as well as sound contours using CARTOSOUND^®^ (Biosense Webster, Diamond Bar, CA, USA) have been utilized but again remain only capable of estimating the location of anticipated ablation. In addition, there is no objective measure of contact with the left atrial tissue similar to that seen with contact force radiofrequency (RF) ablation catheters. To date, we do not have a method to exactly predict and deliver CB lesions on a 3D map similar to with an RF ablation catheter. In this case, we describe how a complete NOCA procedure can be done with anatomically planned and predictable good-contact segmental lesions in order to achieve WACA and PVI in a first pass. To the best of our knowledge, this method, where no attempt was made to achieve PV occlusion, has not been demonstrated previously in conjunction with high-density mapping.

## Case presentation

### Patient history

A 64-year-old woman with no significant past medical history began experiencing recurrent AF episodes over the previous several months. She had 2 prior hospital visits for AF. She was given sotalol but continued to have AF episodes. Her cardiac workup, including echocardiography and laboratory test results, were otherwise unremarkable. After discussion about various treatment options, the patient decided to proceed with AF ablation. We chose the CB modality for AF ablation in this relatively young patient with paroxysmal AF.

### Procedural setup

General anesthesia was used. A transesophageal echo was done to ensure there was no left atrial appendage thrombus. The right and left femoral veins were accessed under ultrasound guidance. Two 8-French sheaths were placed in the right femoral vein, and 7- and 9-French sheaths were placed in the left femoral vein. Heparin was used as per protocol to maintain the activated clotting time at >350 s throughout the procedure. First, using the SL-1 sheath and BRK-1 needle (Abbott, Chicago, IL, USA), transseptal access was gained in a standard fashion with ICE guidance. An Advisor™ HD Grid catheter (Abbott) was then used to create an electroanatomic map of the left atrium (LA). The SL-1 sheath was then exchanged for a Flex sheath (Medtronic, Minneapolis, MN, USA) while leaving the TORAYGUIDE™ guidewire in place (Toray Industries Inc., Tokyo, Japan). The second 8-French sheath was exchanged for the SL-1 sheath. A deflectable decapolar coronary sinus (CS) catheter was advanced into this SL-1 sheath close to the interatrial septum (IAS). The IAS was visualized on ICE. Under ICE guidance, the CS catheter was advanced into the LA with the tip free in the cavity. The SL-1 sheath was advanced over this into the LA. The CS catheter was replaced with the Advisor™ HD Grid catheter, and the sheath was pulled back into the right atrium (RA). The Flex sheath was advanced over the TORAYGUIDE™ guidewire, and the TORAYGUIDE™ guidewire and dilator were removed. The CB was then advanced into the LA.

### Mapping, determination of segmental non-occlusive cryoablation lesion sites, and cryoballoon ablation

**Figure 1:**
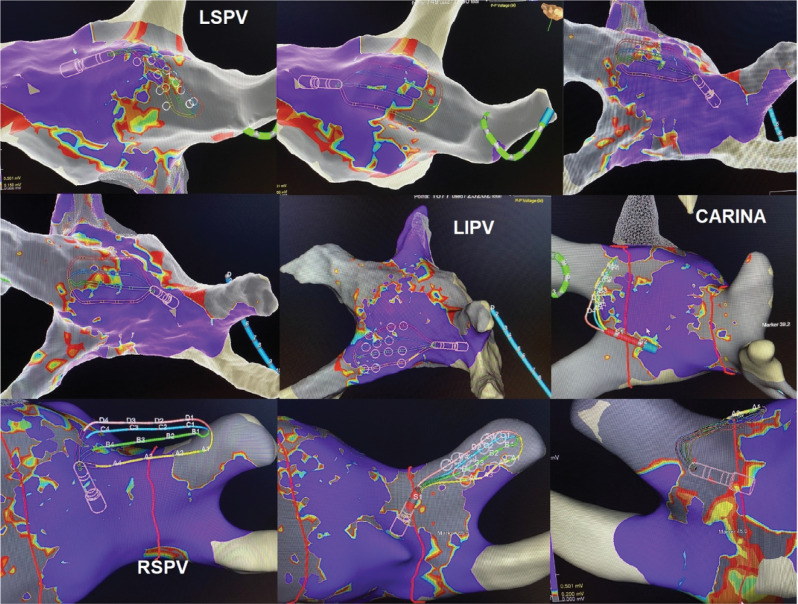
Various positions of the Advisor™ HD Grid catheter for pulmonary vein isolation. Adjusted and aligned with the proximal half of the cryoballoon. *Abbreviations:* LIPV, left inferior pulmonary vein; LSPV, left superior pulmonary vein; RSPV, right superior pulmonary vein.

**Figure 2:**
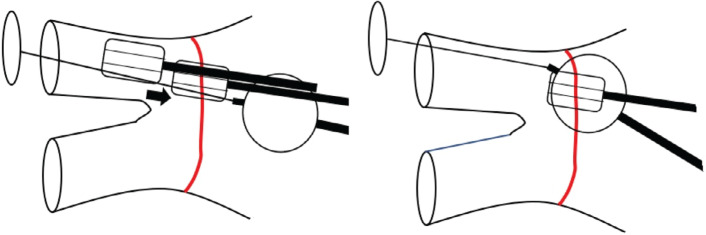
Illustration showing the workflow steps of positioning the HD Grid at the predetermined location on the wide area circumferential ablation line followed by placement of the front half of the cryoballon adjacent to it. This would then be followed by deflection of the cryoballoon toward it and pulling back of the Advisor™ HD Grid catheter before cryoenergy application (not shown here).

**Figure 3:**
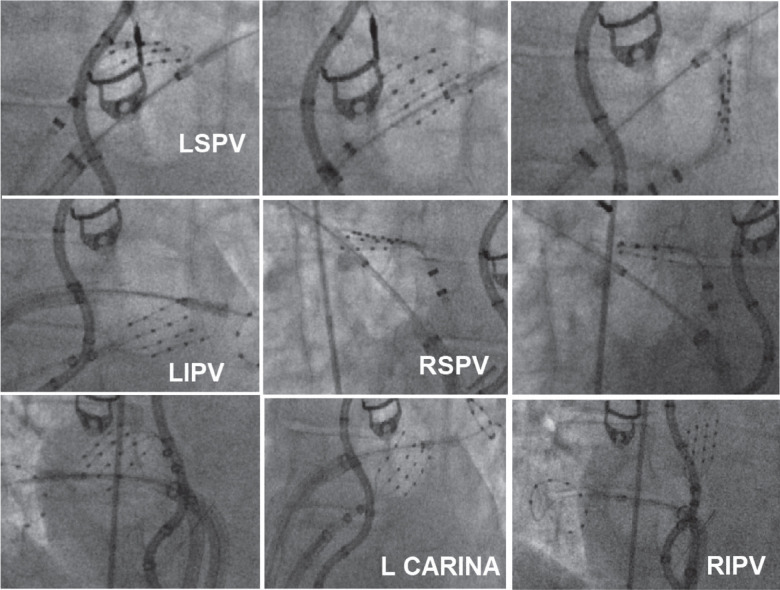
Fluoroscopic images of HD Grid alignment with the proximal half of the cryoballon at various locations on the wide area circumferential ablation lines. *Abbreviations:* LIPV, left inferior pulmonary vein; LSPV, left superior pulmonary vein; RIPV, right inferior pulmonary vein; RSPV, right superior pulmonary vein.

Next, the electroanatomic map of the LA was reviewed, and WACA design lines were drawn to determine the intended site of ablation application. We started with the left superior pulmonary vein (LSPV), which was noted to be the largest vein in this patient. The Advisor™ HD Grid catheter was positioned at the superior part of the posterior aspect of the LSPV on the pre-determined WACA line **(Figures 1 and 2)**. The CB was inflated and advanced toward the LSPV. As we know, the balloon’s front half is the surface where cryoablation occurs.^2^ Our goal was to ensure that the HD Grid and front half of the CB landed at the pre-determined site of the WACA line, as noted in **Figure 2**. The CB and the sheath were then slightly deflected, rotated slightly clockwise, and pushed superiorly to deflect the Advisor™ HD Grid catheter to ensure contact **(Figures 1–3)**. The CB and HD Grid positions were adjusted as needed to achieve this position right on the WACA line with the location guided by the HD Grid to overlap the proximal half of the CB **(Figures 1–3)**. The HD Grid deflection was noted on fluoroscopy images as well as on mapping figures. As this procedure was planned as NOCA, no contrast was used. An LA pressure waveform was available but not monitored due to the non-occlusive nature of the ablation. ICE and color Doppler data were also not monitored due to the same reasons. ICE was used to confirm the relatively proximal position of the CB and correlation with the map.

**Figure 4:**
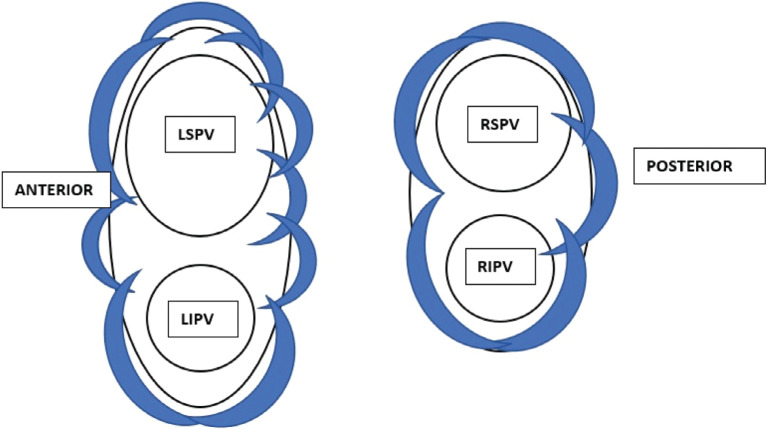
Illustration showing cryoballoon lesions applied to complete WACA lines (see **Table 1** for details). *Abbreviations:* LIPV, left inferior pulmonary vein; LSPV, left superior pulmonary vein; RIPV, right inferior pulmonary vein; RSPV, right superior pulmonary vein.

CB ablation was started, and, at 5 s, the Advisor™ HD Grid catheter was gently pulled back toward the LA off of the CB to avoid inadvertent freezing of the HD Grid. This is important as the safety of freezing the Advisor™ HD Grid catheter together with CB has not been determined by in vitro or in vivo studies. A 120-s lesion was applied. No change was noted in the esophageal temperature with the CIRCA S-CATH temperature probe (Circa Scientific, Englewood, CO, USA). After thawing of the CB, the HD Grid was placed at the site of the ablation to show the location and extent of the NOCA at the intended site, which was anatomically highly predictable. **Figure 1** shows how the extent of the lesion applied at a given location can be immediately assessed by the HD Grid. The information is then used to plan the next lesion with some intentional overlap. These steps were then repeated to complete the pre-determined WACA lines. An additional carina lesion was applied if needed as guided by the voltage **(Figures 1, 2, and 4)**. Other PVs were ablated in a similar fashion **(Figure 4, Table 1)**.

**Figure 5:**
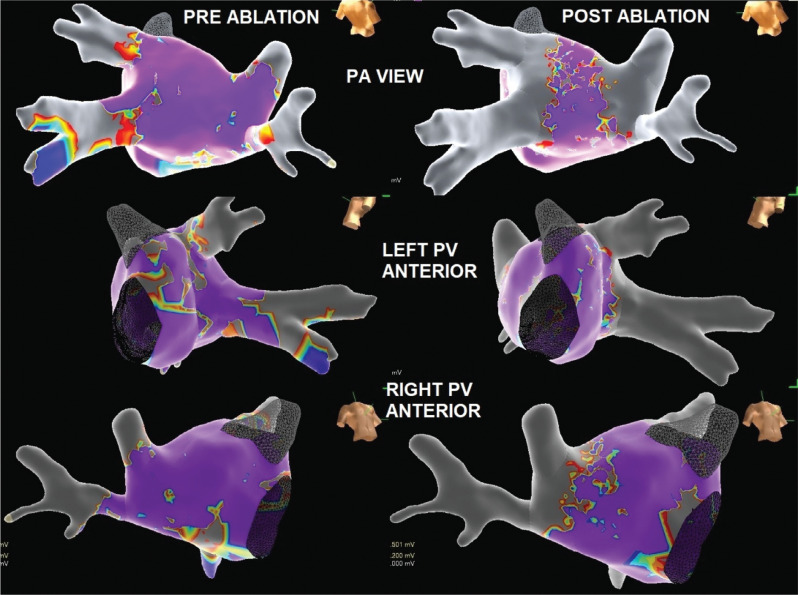
High-density maps created before and after cryoballon ablation using the Advisor™ HD Grid catheter. *Abbreviations:* PA, posteroanterior; PV, pulmonary vein.

First-pass PVI was achieved with a demonstration of the entrance and the exit block immediately evident after the completion of the lesion sets. These were confirmed again at 30 min. Phrenic nerve pacing was used to monitor the function for right-sided PVs. Again, the lesions were very proximal compared to the antrum of the vein, and there was no loss of right phrenic nerve capture. The final voltage map showed WACA **(Figure 5)**. The procedure was then concluded. No significant residual shunt was noted at the site of transseptal access.

## Discussion

CB ablation involves a combination of PV occlusion and additional non-occlusive lesions to achieve PVI.^3,4^ Typically, NOCA is not done if PV occlusion leads to PVI. However, this practice has been evolving.^1^ PV occlusion can be challenging at times. Contrast injections may be required, which expose patients to potential adverse effects of contrast and radiation. Some operators use LA pressure waveform changes to show PV occlusion. This is, however, technique-sensitive and has a learning curve. ICE and the use of color Doppler imaging also allow us to show PV occlusion. However, ICE images of PVs are not always optimal, especially those from the RA. Imaging of PVs from the LA has also been described but not with widespread adoption. As can be seen, there is considerable heterogeneity in achieving and ensuring PV occlusion, and contact of the balloon with the PV ostia can vary between locations. It is often required to deliver additional lesions based on the time to effect and esophageal temperature. Despite all this, PV isolation is often not achieved and NOCA segmental lesions are required due to challenging PV anatomy. Stacked lesions may carry chances of collateral damage to the esophagus or phrenic nerve. It is important to note that none of these techniques consistently accurately predict the site or quality of the lesion at a given PV location. We do not have access to contact force, a drop in impedance, or signal attenuation at the site of ablation as we do in RF catheter ablation.

Lately, there has been growing interest in delivering a NOCA lesion to achieve WACA similar to during RF AF ablation.^1^ Additionally, CB ablation for the posterior LA wall has now been described.^1^ The importance of NOCA lesions is self-evident in achieving WACA for PVI and posterior wall (PW) substrate ablation. The creation of these lesions is mainly guided by fluoroscopy and the estimation of ablation lesion delivery guided by the relative position of the balloon compared to the previous lesion. Sound mapping (CARTOSOUND^®^) is also used to estimate the position of the balloon relative to the PV antrum; however, limitations of ICE imaging quality remain, and this approach requires an operator’s ability to obtain good ICE images. Also, it does not allow pre-selection of the ablation site and does not predict the exact location.

The ideal solution would be to have electrodes on the balloon itself to allow visualization of the CB on the map of the LA. This is, however, not possible, and even if it were possible, it may not necessarily demonstrate true contact such as we observed using this method. After the Advisor™ HD Grid catheter is placed on a desired location of the WACA line, putting the CB’s front half next to it and deflecting it (by clockwise rotation, counterclockwise rotation, pushing up, or pulling down) would deflect the Advisor™ HD Grid catheter over the curve of the CB. Aligning them next to each other and deflecting the Advisor™ HD Grid catheter by the front half of the CB ensures the desired location of the ablation lesions and contact for that part of the WACA line. This step can be repeated along the WACA line, skipping anterior and posterior to avoid stacking lesions on the PW of the LA. This approach shows that a high-definition map can be created and PV anatomy can be completely delineated beforehand. It also gives improved information about the LA voltage compared to that obtained with the Achieve catheter (Medtronic). The ablation lesions can be well planned. The front surface of the CB that actually performs the ablation can be accurately and predictably placed at the intended site of the ablation to achieve a predictable and durable lesion that is not limited by the need to struggle to achieve PV occlusion using various modalities. Technically, the procedure potentially becomes easier due to this.

In addition, we know that 120-s lesions can create good PV ablation.^1,2^ Many times, depending on the size of the vein, PVI is achieved with <4 lesions (right-sided veins were isolated in 2 lesions/vein in this patient). The duration of the lesion being 120–150 s leads to sparing of the esophagus and avoids stacked lesions altogether. Many proximal lesions can be delivered without worrying about CB PV occlusion. Such proximal lesions may further reduce the risk of right phrenic nerve injury. Unlike the limited capacity of the Achieve catheter, a high-definition map created with HD Grid technology allows us to accurately map the voltage with high fidelity. This case achieved first-pass isolation; however, in case PV isolation is not achieved, the Live View feature would accurately allow the localization of PV connection. This can be potentially useful when using the CB for redo AF ablation procedures. Although the PW of the LA was not ablated in this case, the accurate localization and planning of CB ablation lesions on the PW with this method are predictable.

### Limitations

To the best of our knowledge, this is the first case that has described the use of high-density mapping catheter-guided point-by-point segmental NOCA without using any traditional methods of CB ablation, such as contrast injection, pressure waveform, ICE/color Doppler imaging, or fluoro-guided estimated CB ablation location. The wider experience remains to be seen, and this approach is not a substitute for the conventional CB approach or indicated for routine use. The procedure involves additional steps related to second transseptal access and catheter exchanges. In addition, it involves the creation of a workflow that takes some time in getting used to. The cost of the additional Advisor™ HD Grid catheter can also be significant. In addition, long-term results with this approach remain to be seen in a larger clinical trial comparing traditional CB ablation versus complete NOCA. One can potentially argue for using this approach to complement the conventional approach when PVI is not achieved despite the best efforts of the conventional CB applications and additional segmental lesions are needed. This approach can potentially guide operators to the exact locations of the gaps in PVI by voltage mapping as well as other features, such as the Live View, of omnipolar mapping.^5^

## Figures and Tables

**Table 1:** Details of Cryoballon Ablation Applications

Location of CB Ablation Lesion	CB Application Duration (s)	Location of CB Ablation Lesion	CB Application Duration (s)
LSPV—anterior superior	150	RSPV—anterior	150
LSPV—roof	120	RSPV—posterior	150^a^
LSPV—posterior (superior aspect)	120	RIPV—anterior	140
LSPV—posterior (inferior aspect)	120^a^	RIPV—posterior	140^a^
LSPV/LIPV—anterior carina	120	RSPV/RIPV—posterior carina	145
LSPV/LIPV—posterior carina	120		
LSPV/LIPV— posterior carina (superior part)	120		
LIPV—anterior	120		
LIPV—posterior	120^a^		

*Abbreviations:* CB, cryoballoon; LIPV, left inferior pulmonary vein; LSPV, left superior pulmonary vein; RIPV, right inferior pulmonary vein; RSPV, right superior pulmonary vein. ^a^Denotes isolation of the pulmonary vein.
